# Non-GNSS Smartphone Pedestrian Navigation Using Barometric Elevation and Digital Map-Matching

**DOI:** 10.3390/s18072232

**Published:** 2018-07-11

**Authors:** Daniel Broyles, Kyle Kauffman, John Raquet, Piotr Smagowski

**Affiliations:** 1 Air Force Institute of Technology, 2950 Hobson Way, Wright-Patterson AFB, OH 45433-7765, USA; daniel.broyles@us.af.mil (D.B.); kyle.kauffman@afit.edu (K.K.); 2 Military Institute of Armament Technology, 05-220 Zielonka, Poland; smagowskip@witu.mil.pl

**Keywords:** smartphone sensor fusion, particle filter, pedestrian dead reckoning, map-matching

## Abstract

Pedestrian navigation in outdoor environments where global navigation satellite systems (GNSS) are unavailable is a challenging problem. Existing technologies that have attempted to address this problem often require external reference signals or specialized hardware, the extra size, weight, power, and cost of which are unsuitable for many applications. This article presents a real-time, self-contained outdoor navigation application that uses only the existing sensors on a smartphone in conjunction with a preloaded digital elevation map. The core algorithm implements a particle filter, which fuses sensor data with a stochastic pedestrian motion model to predict the user’s position. The smartphone’s barometric elevation is then compared with the elevation map to constrain the position estimate. The system developed for this research was deployed on Android smartphones and tested in several terrains using a variety of elevation data sources. The results from these experiments show the system achieves positioning accuracies in the tens of meters that do not grow as a function of time.

## 1. Introduction

The Global Positioning System (GPS) and other global navigation satellite systems (GNSS) offer unmatched accuracy for outdoor positioning, navigation, and timing (PNT) applications. As a result, the world’s reliance on GNSS has grown significantly in the past decade. However, many applications that depend on GNSS for PNT do so without a backup should GNSS fail [1]. For navigation applications, GNSS are inherently limited to environments with a clear view of the sky and areas that are free of large obstacles that attenuate satellite signals or cause multi-path interference. Furthermore, GNSS are susceptible to both intentional and unintentional interference which can prevent reliable navigation with these systems within affected areas. The inability to navigate in areas where GNSS are either unreliable or unavailable can have severe consequences for many users [2]. Therefore, it is of significant interest to develop technologies that provide reliable navigation where GNSS performance is denied or degraded.

Advancements in micro-electro-mechanical systems (MEMS) technology have given rise to the smartphone as a suitable platform for pedestrian navigation. Smartphones are equipped with GNSS receivers which provide primary positioning information, as well as an array of MEMS sensors that can be used to improve positioning solutions with sensor fusion techniques. Much research has been conducted on the use of smartphones as navigation platforms [3,4,5,6,7,8,9]. However, very few of the technologies proposed in the literature offer navigation solutions that can operate without the aid of external signals, such as GPS or WiFi, or additional hardware, such as high-quality inertial measurement units, antennas, and receivers.

This research expands upon the work completed by Smagowski, Raquet, and Kauffman [10], which demonstrated the feasibility of a non-GNSS pedestrian navigation algorithm that uses only the existing sensors on a smartphone and a digital elevation model (DEM) (i.e., an elevation map). The accuracy achieved by the algorithm was promising; however, the results were obtained by post-processing smartphone data on a desktop computer. Additionally, the algorithm was evaluated using a single DEM source with very fine resolution (2 m post spacing) and a high degree of accuracy (0.3 m root mean square error (RMSE)). To take the proof-of-concept further, this research endeavored to meet the following objectives:

1. Implement the Smagowski et al. algorithm in a real-time smartphone application
2. Characterize the effect of DEM resolution and accuracy on the algorithm’s performance
3. Incorporate design features that increase the robustness of the algorithm

The system developed for this research was deployed on chest-mounted Android smartphones and tested at several locations using a variety of DEM sources. The results from testing showed the system is capable of achieving reliable positioning with errors that do not grow as a function of time. The accuracy of the system is terrain-dependent, but typically stays within tens of meters. For a non-GNSS outdoor pedestrian navigation system, this level of accuracy is adequate for many applications. The system could also be adopted as the base algorithm for other non-GNSS technologies, such as signals of opportunity or magnetic anomaly map-matching approaches, to further enhance position accuracy and reliability in GNSS-denied environments.

## 2. Materials and Methods

The centerpiece of the algorithm used in this research is the particle filter, which is a sequential Monte Carlo method commonly used to solve Bayesian estimation problems for systems with non-linear, non-Gaussian dynamics or measurements [11]. The goal of Bayesian estimation is to recursively estimate the posterior probability density function (PDF) of hidden states in a modeled system based on observations and system inputs. For a non-linear, non-Gaussian system, this PDF is often analytically intractable, which limits the usefulness of many traditional filtering approaches. The particle filter approximates the posterior PDF of the states using a set of weighted samples or particles obtained from a different PDF that is relatively easy to draw from and has the same support as the true density [12]. As the number of particles used in the approximation increases, the estimated PDF approaches the true PDF, and the filter solution approaches the optimal Bayesian estimate in the minimum mean square error (MMSE) sense [13,14]. Specifically, we used the Sequential Importance Resampling (SIR) particle filter variant described in [11].

### 2.1. Algorithm Overview

**Figure 1:**
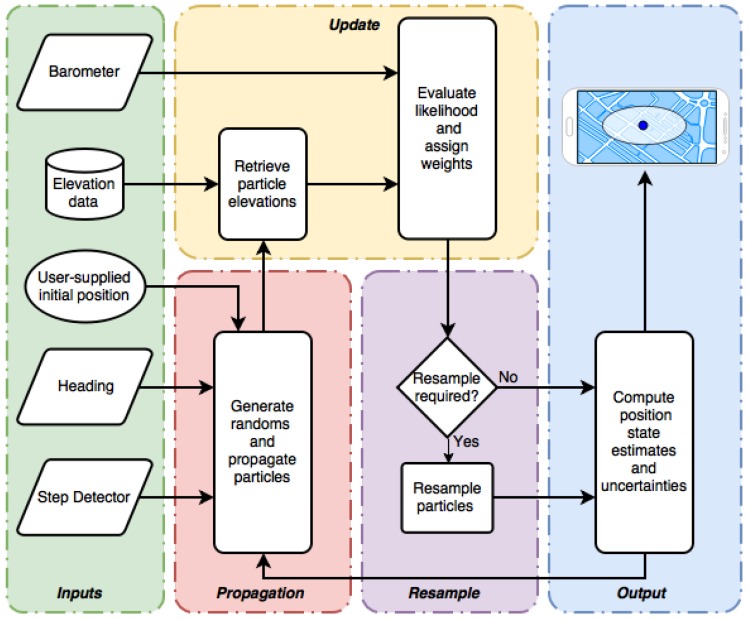
Algorithm functional block diagram.

Figure 1 shows a functional block diagram of the particle filter algorithm used in this research, which was adapted from [10]. The algorithm begins in the input stage (green) with the user providing the starting location, which is assumed to be accurate to within 5 m. This position information is used to initialize the particle filter. Once initialized, the system remains idle until a step is detected (see Section 2.5.1), at which point the particles are propagated using the current heading input and randomly generated values for step length deviations and biases (see Equation (4)). The particle weights remain unchanged during propagation.

When a barometric elevation measurement is available, the elevation corresponding to each particle’s location is extracted from the DEM in the update stage (yellow). A likelihood function (Equation (7)) is then evaluated which assigns particle weights based on how closely each particle’s elevation matches the realized measurement. Particles with elevations that closely match the barometric elevation are highly weighted and are therefore more influential in the final position solution, whereas low-weighted particles are less likely and contribute very little to the solution.

After the update stage, a resampling scheme is executed, if needed, which replaces particles with negligible weights with highly weighted particles (see Section 2.4.1). This effectively kills off particles that are likely carrying incorrect state estimates, and adds more particles to areas within the state space which are more likely to be correct. Lastly, the position estimate and associated uncertainty is computed from the weighted particles (see Equations (8) and (9)) and displayed on the smartphone’s display as a location marker and error ellipse in the output stage (blue). The particles used for this computation are either continually updated as new barometer measurements become available or are propagated again once the next step is detected.

### 2.2. Stochastic Model and Particle Filter Formulation

In this section, a state space representation of the navigation system is presented which mathematically models pedestrian motion, system inputs, barometer sensor measurements, and random noise. This stochastic model serves as the foundation for the particle filter and provides insight into the parameters that affect filter performance. The generic parameters introduced in this section are fully defined in Section 2.5 which covers filter tuning and sensor data conditioning.

The states of interest for the system include two position states and two bias states. The two position states, *x* and *y*, represent the distances from the starting location (provided by the user) in the east and north directions, respectively. The heading bias state, $b_{\theta }$, represents the time-varying error in the system’s heading input, and is modeled as a first-order Gauss–Markov (FOGM) process [15]. The barometric elevation bias state, $b_{b}$, represents the time-varying error in the barometric elevation measurement, and is also modeled as a FOGM process. The state vector for the system is

(1) $$x={\left[\begin{array}{cccc} x & y & b_{\theta } & b_{b} \end{array}\right]}^{T}$$

The individual particles are composed of two attributes, namely, a state mean estimate (${\hat{x}}_{k}^{i}$ is the 4 × 1 mean vector for the *i*th particle at the *k*th step) and a weight ($\omega_{k}^{i}$). The mean vector and covariance matrix for the entire particle set at the *k*th step are denoted by ${\hat{x}}_{k}$ and $P_{k}$, respectively. Initially, particles are drawn from a normal distribution, with a mean (${\hat{x}}_{0}$) corresponding to the initial position and bias state estimates, and a covariance ($P_{0}$) corresponding to the uncertainty of those estimates. The number of particles used is denoted by N. Upon initialization, the particles are equally weighted, such that $\omega_{k}^{i}=\frac{1}{N}$ for i=1,…N. Once initialized, the particle filter’s state mean and covariance estimates are formed recursively in two stages: propagation and measurement update.

### 2.3. State Propagation

With each step taken, the state mean vector is propagated by a non-linear state transition function of the prior state mean vector, heading (θ), average step length ($\bar{l}$), time passed since the last propagation (Δt), and process noise ($\eta_{k}$). The generic equation for propagating between consecutive steps taken is

(2) $${\hat{x}}_{k}=f\left({\hat{x}}_{k-1},\theta_{k},\bar{l},\Delta t_{k},\eta_{k}\right)$$

This model assumes that any uncertainty in the *x* and *y* states is strictly a function of other sources of uncertainty, namely, heading and step length. The process noise vector for the model, $\eta_{k}$, is given by

(3) $$\eta_{k}={\left[\begin{array}{ccc} \delta_{l_{k}} & w_{\theta_{k}} & w_{b_{k}} \end{array}\right]}^{T}$$

where

- $\delta_{l_{k}}$ represents random deviations in step length; $\delta_{l_{k}}∼U\left(-\sigma_{l},\sigma_{l}\right)$
- $w_{\theta }$ and $w_{b}$ are driving white noises for the FOGM states; $w_{\theta }∼N\left(0,\frac{2\sigma_{\theta }^{2}}{\tau_{\theta }}\right)$ and $w_{b}∼N\left(0,\frac{2\sigma_{b}^{2}}{\tau_{b}}\right)$

The step length deviation ($\delta_{l_{k}}$) is modeled as a uniformly-distributed random variable, as was the case in the Smagowski et al. algorithm [10]. This assumption was made in order to bound the total step length and avoid unrealistic deviations, as would be the case in the tails of a normal distribution. The standard deviations ($\sigma_{\theta },\sigma_{b}$) and time constants ($\tau_{\theta },\tau_{b}$) are tunable FOGM parameters that control the magnitude and frequency content of the modeled biases [15]. The process noise is assumed to be independent.

When a step is detected, the particles are propagated independently using the following equation:

(4) $$\overset{{\hat{x}}_{k}^{i}}{\overset{︷}{\left[\begin{array}{c} x_{k}^{i}\\ y_{k}^{i}\\ b_{\theta_{k}}^{i}\\ b_{b_{k}}^{i} \end{array}\right]}}=\overset{f({\hat{x}}_{k-1}^{i},\theta_{k},\bar{l},\Delta t_{k},\eta_{k})}{\overset{︷}{\left[\begin{array}{cccc} 1 & 0 & 0 & 0\\ 0 & 1 & 0 & 0\\ 0 & 0 & e^{-\frac{1}{\tau_{\theta }}\Delta t_{k}} & 0\\ 0 & 0 & 0 & e^{-\frac{1}{\tau_{b}}\Delta t_{k}} \end{array}\right]\left[\begin{array}{c} x_{k-1}^{i}\\ y_{k-1}^{i}\\ b_{\theta_{k-1}}^{i}\\ b_{b_{k-1}}^{i} \end{array}\right]+\left[\begin{array}{c} \left(\bar{l}+\delta_{l_{k}}\right)\sin\left(\theta_{k}+b_{\theta_{k-1}}^{i}\right)\\ \left(\bar{l}+\delta_{l_{k}}\right)\cos\left(\theta_{k}+b_{\theta_{k-1}}^{i}\right)\\ w_{\theta_{k}}\\ w_{b_{k}} \end{array}\right]}}$$

In Equation (4), the first two rows propagate the position states via pedestrian dead reckoning (PDR), which computes a new position from the previous position given the current step length and heading inputs [4]. In this case, the system inputs are the average step length plus a random deviation and the current heading reported by the smartphone sensors (see Section 2.5.2) plus the heading bias estimate from the prior step. The last two rows in Equation (4) propagate the bias states according to the standard FOGM dynamics, as defined in [15]. Note that, while the process noises associated with the FOGM biases are additive with respect to the states, the noise in the step length is multiplicative.

### 2.4. Measurement Update

The measurement model for the system captures how the states at a given step epoch are related to the barometric elevation measurements, denoted by *z*, observed at that same epoch. For an individual particle, this relationship is represented by a non-linear function that maps a particle’s position to its corresponding elevation, taking the particle’s barometric elevation bias estimate into account:

(5) $$\begin{array}{cc} z_{k} & =h\left({\hat{x}}_{k}^{i}\right)+v_{k} \end{array}$$

(6) $$\begin{array}{cc} h({\hat{x}}_{k}^{i}) & =\varepsilon \left(x_{k}^{i},y_{k}^{i}\right)-b_{b_{k}}^{i} \end{array}$$

where

- $z_{k}$ is the barometric elevation measurement at step *k*
- $v_{k}$ is the additive white Gaussian measurement noise; v∼N(0,R)
- ε($x_{k}^{i}$,$y_{k}^{i}$) is a function that returns the elevation at the location ($x_{k}^{i}$,$y_{k}^{i}$)

In practice, the true elevation returned from ε ($x_{k}^{i}$, $y_{k}^{i}$) is not directly available but is instead inferred from an interpolated DEM. This interpolated elevation is prone to error due to inaccuracies in the underlying data and interpolation error (the latter being especially true for DEMs with coarse resolution). Since the DEM is not a perfect representation of the true elevation, another term should exist in Equation (6) which models DEM error. These errors were not explicitly modeled in our implementation because doing so would significantly increase the number of particles required. Additionally, the barometric elevation bias state and measurement noise terms were able to account for these errors adequately in most cases.

With each barometric elevation measurement, the particle weights were recursively updated by determining the likelihood of the measurement given the particle’s current state estimate. The likelihood was calculated from a Gaussian PDF centered at $h({\hat{x}}_{k}^{i})$ with covariance R, evaluated at the measurement $z_{k}$. The particle weights from the prior step were then multiplied by the output of the likelihood function, resulting in the updated particle weights. For an individual particle, the update equation was

(7) $$\omega_{k}^{i}=\omega_{k-1}^{i}\exp\left(-\frac{1}{2}{\left[z_{k}-h\left({\hat{x}}_{k}^{i}\right)\right]}^{T}R^{-1}\left[z_{k}-h\left({\hat{x}}_{k}^{i}\right)\right]\right)$$

This method rewards particles whose DEM elevations closely match the measured barometric elevation and penalizes particles whose position estimates are less likely to be based on the barometric elevation measurement.

Lastly, the particle filter’s state estimate and covariance were reconstructed from the particles by computing the weighted sample mean, ${\hat{x}}_{k}$, and the sample covariance, $P_{k}$, as follows:

(8) $$\begin{array}{cc} {\hat{x}}_{k} & =\sum_{i=1}^{N}\omega_{k}^{i}{\hat{x}}_{k}^{i} \end{array}$$

(9) $$\begin{array}{cc} P_{k} & =\sum_{i=1}^{N}\omega_{k}^{i}\left({\hat{x}}_{k}^{i}-{\hat{x}}_{k}\right){\left({\hat{x}}_{k}^{i}-{\hat{x}}_{k}\right)}^{T} \end{array}$$

The position states from this computation were then presented to the user on the system’s map display, accompanied by an ellipse representation of $P_{k}$, which corresponds to the estimate uncertainty.

#### 2.4.1. Resampling

One problem common to particle filtering is the condition known as particle degeneracy, in which only a few particles carry non-negligible weights. Since these low-weighted particles carry no useful information, the computational resources spent on these particles are essentially wasted. The SIR particle filter addresses this issue by occasionally resampling the particles, such that low-weighted particles are replaced with highly-weighted particles. The system developed for this research uses the multinomial resampling scheme presented in [16]. After each measurement update, the approximate number of effective particles, N${}_{\mathrm{eff}}$, was calculated using:

(10) $$N_{\mathrm{eff}}\approx \sum_{i=1}^{N}\frac{1}{{\left(\omega_{k}^{i}\right)}^{2}}$$

Resampling is triggered only when $N_{\mathrm{eff}}$ drops below a threshold of 0.5, indicating only 50% of the particles carry useful information.

### 2.5. Data Conditioning and Filter Tuning

#### 2.5.1. Step Detection and Step Length Estimation

Step detection was implemented using Android’s stock step detector sensor. This sensor triggers a time-stamped event when a step is detected from walking, running, or walking up stairs. The sensor has a latency of less than 2 s, making it well-suited for applications that track the number of steps in real-time, such as PDR systems [17].

The average step length, $\bar{l}$, was computed using the following method found in [7]:

(11) $$\bar{l}=c_{1}h_{\mathrm{user}}$$

where $c_{1}$ is a gender specific constant (0.415 for males, 0.413 for females) and $h_{\mathrm{user}}$ is the height of the user in meters. In the propagation stage, this average step length is summed with a random step length deviation drawn from a uniform distribution, $\delta_{l}$, to account for uneven step lengths. The bounds associated with $\delta_{l}$ were tuned to $\sigma_{l}$ = 0.15 m (6 in).

#### 2.5.2. Heading Input and Heading Bias

The system’s heading input was implemented using Android’s rotation vector sensor, which fuses the MEMS accelerometer, gyroscope, and magnetometer outputs to estimate the smartphone’s orientation in the world frame [17,18]. The output from the rotation vector sensor was converted into a true north-referenced heading by adding the appropriate declination angle from a look-up table. The true north-referenced heading was used as the heading input, $\theta_{k}$, in Equation (4).

The heading bias state FOGM parameters were tuned using observations from a wide range of datasets collected for this research. The reference heading bias was derived by subtracting $\theta_{k}$ from the heading based on the GPS data. In most cases, the reference heading biases observed were frequently changing and were bounded by $\pm 15^{\circ }$. Therefore, the heading bias state was tuned such that $\sigma_{\theta }$ = $15^{\circ }$ and $\tau_{\theta }$ = 60 s.

#### 2.5.3. Barometric Elevation Bias and Measurement Noise

The barometric pressure sensors equipped in the smartphones used in this study are fairly sensitive with a data resolution of 0.16 Pa which equates to less than 10 cm of altitude [19]. In order to tune the FOGM parameters associated with the barometric elevation bias state, barometric elevation measurements from the collected data were compared with the corresponding DEM elevations at the GPS positions reported during the collection. The difference between these values served as the reference bias, and the tuning parameters were chosen based on this reference. For the majority of DEM sources used, tuning the barometric elevation bias such that $\sigma_{b}$ = 5 m and $\tau_{b}$ = 3600 s resulted in an adequate representation of the reference bias. For the Shuttle Radar Topography Mission (SRTM) DEM, an alternate tuning of $\sigma_{b}$ = 20 m and $\tau_{b}$ = 1200 s was used to account for the inaccuracies present in the DEM and to avoid filter divergence issues (the accuracy and resolution specifications of the DEMs used in this research are covered in Section 2.6.3).

The measurement noise variance, R, works in concert with the barometric elevation bias state to account for barometer sensor noise; however this parameter also accounts for errors in the underlying elevation data. Intuitively, R can be thought of as the level of confidence in how well the realized measurements will match the elevation data. Through trial and error, this value was tuned to R = 16 $m^{2}$, which yielded reasonable performances for DEMs with 0.5, 2, and 10 m resolutions. For DEMs with 30 m resolution, an alternate tuning of R = 160 $m^{2}$ was needed to achieve a reliable performance.

### 2.6. Experiment Set Up

#### 2.6.1. Data Collection

**Figure 2:**
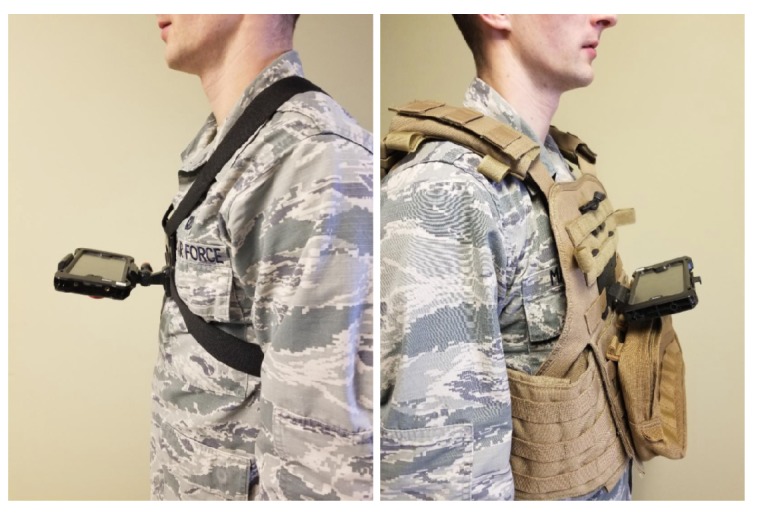
Smartphone Chest Mounts: velocity clip (**left**), juggernaut case (**right**).

Data collection consisted of walking routes with chest-mounted smartphones running the application developed for this research. The chest mounts used are shown in Figure 2. The smartphone models tested include the Samsung Galaxy S7, Motorola Nexus 6, and LG Nexus 5X. In addition to monitoring the real-time display during the experiments, the sensor data was recorded and replayed through an Android Studio-emulated smartphone configured with the same software but loaded with a different DEM. This was repeated for each DEM source acquired for this research, allowing for a comparison of the algorithm’s performance for a given route with respect to DEM accuracy and resolution.

While the emulator itself is a simulation, the results obtained via emulator were considered equivalent to real data for the following reasons. First, the code executed in the emulator was identical to that in the smartphone and was operating on the same sensor data obtained from the real-world experiments. Second, for a specific route/DEM combination, there was no discernible performance difference in the results obtained via emulator versus those obtained from a smartphone. Lastly, the number of particles used in the emulator was intentionally restricted to reflect the processing limitations of smartphones. The smartphone models tested for this research were able to handle up to 500 particles without any significant lag in the results. Therefore, all emulator-generated results supporting conclusions about the performance of the system were conducted with only 500 particles. This number only reflects the limitation of the system in its current implementation, since there are many ways to optimize the software that would allow for more particles to be carried.

#### 2.6.2. Performance Metrics

The performance of the system was quantified by the distance root mean square (DRMS) error with respect to GPS (recorded for comparison only, not used in the algorithm), given by

(12) $$\mathrm{DRMS}=\sqrt{\frac{\sum_{k=1}^{n}{\left(d_{k}\right)}^{2}}{n}}$$

In Equation (12), *n* is the number of steps taken during the route, and $d_{k}$ is the Euclidean horizontal distance measured from the interpolated GPS position to the filter-estimated position at the *k*th step. In two cases, the smartphones were configured with airplane mode turned on and location services turned off for all or some of the route. This was to simulate a GNSS-denied environment. As a result, the DRMS values reported for those trials were only calculated at step epochs during which GPS was turned on.

#### 2.6.3. Elevation Data Sources

The five DEMs acquired for this research are listed in Table 1. The tags in the second column are used to differentiate results obtained using these DEMs throughout this article. All of the sources listed are freely available online, with the exception of the VDTM0.5 which was donated courtesy of Vricon. The availability of the DEMs are also listed in Table 1, which was an important consideration for this research due to the algorithm’s dependency on elevation data. While only the SRTM and Vricon are globally available, many countries have agencies that provide products and services similar to the U.S. Geological Survey (USGS). Also, the popularity of lidar has grown in recent years, and efforts are underway to create an open-source global lidar database as these products become available [20]. The last two columns of Table 1 list the resolution and the accuracy specifications for the DEMs. The resolutions range from very coarse (30 m post spacing) to very fine (0.5 m post spacing). The Ohio Statewide Imagery Program (OSIP) DEM is significantly more accurate than the rest of the DEMs, whereas the USGS and Vricon DEMs have similar accuracy specifications, and the SRTM DEM is the least accurate by a wide margin.

#### 2.6.4. Testing Locations

In order to better understand the effects of terrain on the system’s performance, testing locations were categorized based on their respective elevation profiles. Routes consisting of less than 10 m of elevation change throughout the route were categorized as flat terrain. Routes consisting of steadily changing elevation with more than 10 m of total elevation change were categorized as slanted terrain (i.e., steep hills). Routes consisting of high spatial-frequency changes in elevation were categorized as varied terrain. There were six distinct testing locations, two of each category (flat, slanted, and varied). Experiments were conducted outdoors under a variety of weather conditions, including sub-zero air temperatures, high wind speeds, snow, and rain.

## 3. Results

### 3.1. Flat Terrain Performance

Table 2 shows the resulting positioning errors observed in flat terrain for each DEM. The third column shows the dead reckoning (DR) case, obtained from propagating the model without barometric elevation updates, for comparison. System performance was satisfactory for all cases when using VDTM0.5, OSIP2, USGS10, and USGS30, which showed significant reductions in error compared to the DR case. The performance when using USGS10 was best in two of the four cases. As expected, the performance when using SRTM30 was worse than the rest of the DEMs.

**Figure 3:**
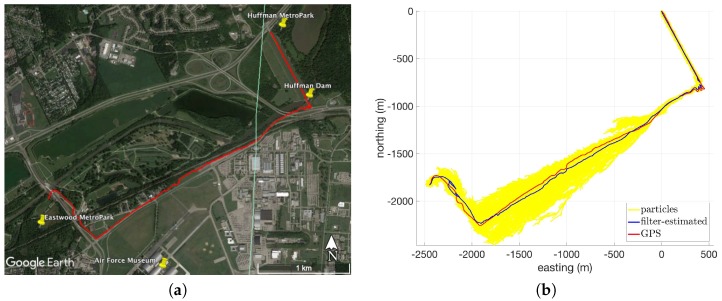
Overhead views of Route 2: (**a**) Google Earth satellite imagery [26]; (**b**) Global Positioning System (GPS) and filter-estimated paths in local level coordinate frame (using USGS10).

Detailed results from Route 2 in Table 2 using USGS10 are presented below to further demonstrate the performance of the system on flat terrain. Figure 3a shows an overhead view of Route 2. This route consists of a pedestrian and biking path that connects two parks in the greater Dayton, Ohio area and crosses over the Huffman Dam bridge and under two highway overpasses near the Eastern portion of the route. The filter-estimated and GPS routes from this experiment are plotted in Figure 3b. The particles (yellow) are concentrated in the first leg of the route but spread out significantly in the second leg of the route. This happens because the second leg is very flat, with approximately 2 m of elevation change over a 1.4 km distance and very little diversity in the surrounding terrain. In effect, the reduced amount of spatial information available to the particle filter in the second leg of the route allows particles to survive longer without being killed off during resampling. In the third leg, the particle filter tracks two potential solutions and eventually settles on the correct one before the route is terminated.

**Figure 4:**
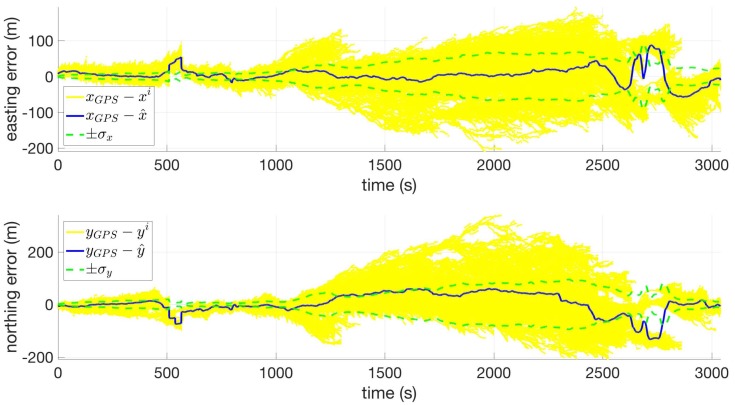
Easting (**top**) and northing (**bottom**) coordinate errors versus time for Route 2 using USGS10.

Figure 4 shows the estimate error versus time for the position states. In general, the error stays within the filter-predicted standard deviation approximately 63.8% of the time, which is an indication that the filter is tuned appropriately. Additionally, this figure more clearly shows the growing uncertainty in the second leg as well as the filter tracking two solution in the third leg.

### 3.2. Slanted Terrain Performance

Table 3 summarizes the performance of the system in slanted terrain for each DEM. In general, the performance of the system when using VDTM0.5, OSIP2, USGS10, or USGS30 was satisfactory. The performance was best when using OSIP2 in four of the seven cases. The performance for Route 5 when using VDTM0.5 was exceptional, as the 14.8 m DRMS error was the lowest recorded for any dataset. Another notable feature in this table is that USGS30 performance was generally very good and outperformed the other DEMs in a few cases. This was not the case in Table 2, suggesting this result can be partially attributed to the increased spatial information available in the slanted terrain. It should also be noted that the DRMS errors observed when using USGS10 were the same (within 5 m) or better than OSIP2 in five of the seven cases. As expected, the SRTM30 performance was significantly worse than the rest of the DEMs, but was still better than DR for all but one case (this particular case was caused by particle starvation, that is, adding more particles resulted in acceptable performance).

**Figure 5:**
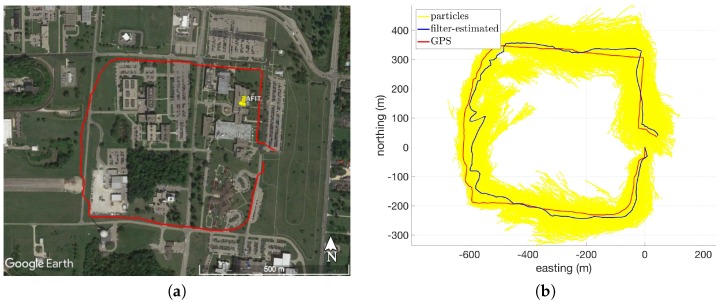
Overhead views of Route 8: (**a**) Google Earth satellite imagery [27]; (**b**) GPS and filter-estimated paths in local level coordinate frame (using VDTM0.5).

Figure 5a shows the overhead view of Route 8, and Figure 5b shows the GPS and filter-estimated routes for this dataset when using VDTM0.5. This route consists of a square path along a relatively steep hill near the Air Force Institute of Technology campus and covers a distance of 2.1 km with approximately 30 m of elevation change throughout the route. Looking at Figure 5a, there is a noticeable deviation in the filter-estimated path in the Western leg of the route. This is caused by a short-duration heading error of approximately 30${}^{\circ }$, which is twice the magnitude of the modeled heading bias (recall $\sigma_{\theta }=15^{\circ }$ from Section 2.5.2). However, the filter is able to fully recover and tracks the true path accurately for the remainder of the route.

**Figure 6:**
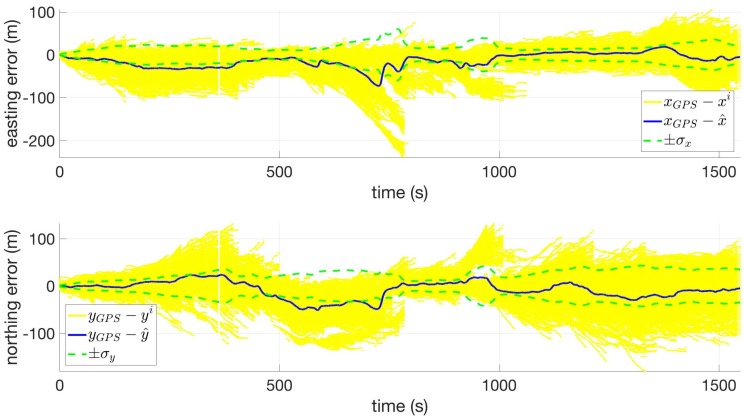
Easting (**top**) and northing (**bottom**) coordinate error versus time for Route 8 using VDTM0.5.

Figure 6 shows the estimate errors for the position states. The errors in these states are mostly bounded by the filter-predicted standard deviation, and this is especially true in the second half of the route. An interesting feature can be seen around t = 800 s, at which point the filter resamples, effectively killing off erroneous particles and locking onto the correct solution. Also, note that the magnitude of the filter-predicted standard deviations for the position states are less than ±50 m for most of the route, whereas these magnitudes reach as much as ±100 m for the flat terrain route shown in Figure 4. This effect can be attributed to the variation in the slanted terrain, which allows the particle filter to resample particles more frequently and limits the uncertainty growth.

### 3.3. Varied Terrain Performance

Table 4 summarizes the system’s performance for routes consisting of varied terrain. Performance in these experiments was generally satisfactory when using OSIP2, USGS10, and USGS30 DEMs. Performance was best when using the OSIP2 DEM in three of the four routes; however, the differences in performance when using the OSIP2 versus USGS10 were minor (within 5 m). The SRTM30 performance was better than DR in most cases, except for Route 14, in which adjusting the baro bias tuning yielded acceptable results but only for that specific dataset.

**Figure 7:**
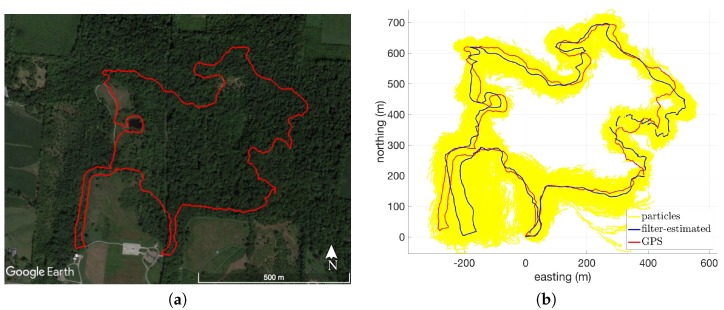
Overhead views of Route 14: (**a**) Google Earth satellite imagery [28]; (**b**) GPS and filter-estimated paths in local level coordinate frame (using USGS30).

Figure 7a shows an overhead view of Route 14, which was collected at the Charleston Falls Preserve in Tipp City, OH. The route consists of mostly forested terrain with very diverse terrain features. Figure 7b shows the GPS and filter-estimated paths for this dataset when using USGS30. There is some disagreement in the two paths shown in Figure 7b in the Southwestern segment which corresponds to the flattest portion of the route. There is also some deviation from the true path seen in the Southeastern segment. During this portion of the route, the pedestrian walked down a set of man-made stairs that lead into a steep ravine at the base of the park’s centerpiece waterfall. Inside the ravine, the self-reported accuracy of the smartphone’s GPS was as much as 15 m (68% confidence radial horizontal accuracy), most likely caused by multi-path interference in the ravine.

**Figure 8:**
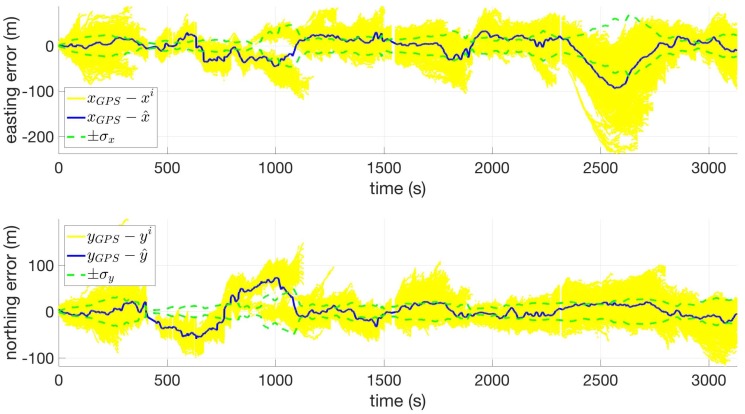
Easting (**top**) and northing (**bottom**) coordinate error versus time for Route 14 using USGS30.

Figure 8 shows the estimate error for the position states for Route 14 using USGS30. The interval from t = 400 s to t = 650 s corresponds to the ravine area, in which the calculated error is inaccurate due to the aforementioned GPS multi-path interference. Aside from the ravine, Figure 8 shows that the estimate error for the two position states is mostly bounded by the filter-predicted standard deviation. At t = 2500 s, the pedestrian is traveling through the relatively flat portion in the southwest segment of the route, and the uncertainty in the *x* state grows as a result until t = 2800 s, at which point the filter clamps down on the correct solution. Also note that, for most of the route, the filter-predicted standard deviations are much tighter than the flat and slanted cases, which, again, can be attributed to the large amount of spatial information available within the terrain.

**Figure 9:**
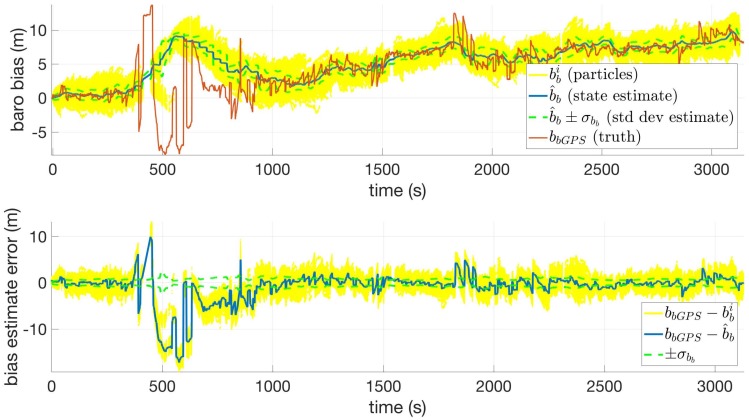
Baro bias (**top**) and baro bias estimate error (**bottom**) versus time for Route 14 using USGS30.

One interesting feature of the Route 14 dataset is that it contains the largest barometric elevation drift observed throughout this research. Figure 9 shows this bias, which reaches approximately 10 m in magnitude over the duration of the route. The erratic behavior in the red line is an effect of GPS multi-path in the ravine. Dismissing the ravine area, the filter appears to have high observability of the baro bias state and tracks the bias within a few meters, as shown in the bottom plot of Figure 9.

### 3.4. Performance Summary

Table 5 summarizes the performance in each terrain type category and for each DEM. The values shown are the RMS of the DRMS errors from Table 2, Table 3 and Table 4, and the last row is the total RMS of these values for each DEM. Looking at the last row of Table 5, the overall performance of the system when using VDTM0.5, OSIP2, USGS10, and USGS30 was satisfactory, with errors less than 61 m across all terrain types. The performance when using VDTM0.5, OSIP2, and USGS10 was very good, with errors less than 48 m across all terrain types. The performance when using SRTM30 was two to three times worse than the rest of the DEMs; however, the DRMS errors observed were still better than in the DR case.

In general, it can be seen from Table 5 that performance was best in slanted terrain in terms of the observed DRMS errors. However, it is important to note that the errors observed from the DR case in slanted terrain were coincidentally the lowest of the three terrain types. In terms of the improvement in accuracy compared to the DR case, the varied terrain results were significantly better.

The differences in the overall performance between the 10 m or less post spacing DEMs was negligible (within 2 m of each other). Additionally, the USGS30 performance was significantly better than the much less accurate SRTM30. This suggests that DEM accuracy is a more significant factor than the resolution.

## 4. Discussion

The results from testing indicate the system is capable of achieving positioning accuracies in the tens of meters, with positioning errors that do not grow as a function of time. The accuracy of the system depends on the elevation diversity of the terrain and the accuracy of the elevation map, and is therefore not directly related to the distance traveled. Positioning errors less than 48 m DRMS can be achieved using DEMs with 10 m post spacing or less, provided the DEM’s accuracy is 3 m (RMSE) or less. The system’s performance was adequate (less than 61 m DRMS error) when using the USGS’s 30 m post spacing DEM. Of the DEM sources tested, Vricon, OSIP, and USGS are all viable options. Marginal accuracy improvements were observed when using NASA’s 30 m SRTM DEM for most of the datasets; however, in some cases, filter tuning issues and particle starvation resulted in unsatisfactory performances.

One aspect of performance not captured by the tables presented in this article is the filter-predicted uncertainty associated with the position estimates. As discussed in Section 2.5, alternate filter tunings were needed for the 30 m DEMs to account for the resolution and errors in these datasets. The effect of using the more pessimistic tuning was that filter-predicted standard deviations were much larger than when using the tuning for DEMs with finer resolution. This was mostly due to the increased value of R. For the SRTM datasets, the alternate barometric elevation bias tuning also contributed to this increased uncertainty. More work is needed to investigate alternative methods for improving the performance of the system when using SRTM30, such as increasing the number of particles or using another set of tuning parameters.

The system operates in near real-time, and there was no perceptible initialization time or time lag in the results observed during operation. There were some cases in which the filter became overconfident and falsely locked onto an incorrect positioning solution. This was often caused by a variety of factors, including modeling error (i.e., step lengths that were much shorter than the model allows), weather effects (high wind speeds inducing fluctuations in the barometer), and similarities in the elevation profiles of the correct and incorrect paths. While beyond the scope of this article, a residual monitoring subroutine was implemented to detect and cope with these situations which effectively aided the filter in recovering the correct position.

The primary advantage of the system presented in this article over other non-GNSS outdoor smartphone positioning systems is that it is self-contained, that is, since the system requires no external reference signals or additional equipment, it is lightweight and immune to jamming. However, environmental interference can negatively affect the system’s performance, specifically, through the presence of man-made sources of magnetic fields, such as buildings made of steel or power transmission lines which can induce magnetometer biases that are larger than the model allows (as was the case in short-duration heading disturbance from Route 8 discussed in Section 3.2). Although the data presented in this article was not collected in dense urban canyons, it is reasonable to assume that the system’s performance could suffer in these environments. Future work should examine the system’s performance in areas densely populated with man-made structures.

Another area of improvement for the system presented in this article is the step length model, which is admittedly simplistic and could be a significant source of positioning error. Future work should consider more sophisticated techniques, such as that used in reference [29], which proposed an empirical model based step frequency, user height, and acceleration amplitude, or reference [30], which proposed using GPS measurements to estimate a set of gait parameters for a specific user (for our purposes, these parameters could be learned and stored prior to using the system in a GPS-degraded environment).

## Figures and Tables

**Table 1:** Digital elevation model (DEM) source comparison.

Source	Tag	Method	Availability	Resolution (m)	Accuracy
SRTM [21]	SRTM30	satellite-based, radar	global	30	≤16 m 90% conf.
USGS. [22]	USGS30	multi-source	U.S.	30	≤2.4 m RMSE
USGS [23]	USGS10	multi-source	U.S.	10	≤2.4 m RMSE
OSIP [24]	OSIP2	lidar flyover	Ohio	2	≤0.3 m RMSE
Vricon [25]	VDTM0.5	satellite-based, imagery	global	0.5	≤3 m 90% conf.

**Table 2:** Flat terrain performance by DEM source.

Route	Distance (m)	DRMS Position Error (m)					
		DR	VDTM0.5	OSIP2	USGS10	USGS30	SRTM30
1	2044.3	89.2	33.6	36.7	53.3	33.1	70.3
2	4788.4	245.3	83.7	63.4	45.1	68.2	142.7
3	3215.3	105.7	29.6	25.6	27.5	34.4	104.9
4	3214.6	108.2	77.3	76.9	59.6	96.7	116.5

**Table 3:** Slanted terrain performance by DEM source.

Route	Distance (m)	DRMS Position Error (m)					
		DR	VDTM0.5	OSIP2	USGS10	USGS30	SRTM30
5	2128.8	72.1	14.8	24.6	28.2	23.4	26.9
6	2199.4	86.0	17.8	16.2	17.2	24.6	82.4
7	2185.8	67.4	44.8	36.4	52.1	34.9	85.6
8	2172.8	130.6	28.8	30.7	41.9	51.0	106.8
9	1241.1	59.6	-	25.4	22.6	33.8	54.9
10	1362.3	39.4	27.7	23.9	23.9	29.7	39.5
11	2221.6	104.0	-	67.5	69.2	68.2	99.0

**Table 4:** Varied terrain performance by DEM source.

Route	Distance (m)	DRMS Position Error (m)					
		DR	VDTM0.5	OSIP2	USGS10	USGS30	SRTM30
12	1754.2	230.7	-	28.6	31.6	82.2	163.6
13	5559.3	135.3	-	52.5	58.6	67.6	120.0
14	4169.9	156.1	-	19.2	20.4	62.1	163.6
15	5453.4	389.3	-	68.7	67.7	75.8	363.4

**Table 5:** Performance Summary.

Terrain	DR	RMS of DRMS Position Error (m)				
		VDTM0.5	OSIP2	USGS10	USGS30	SRTM30
flat	150.7	61.2	54.6	47.9	63.8	111.7
slanted	84.6	28.8	35.6	40.4	40.8	76.2
varied	248.7	-	46.5	48.6	72.3	253.1
all terrains	174.8	47.8	46.2	45.8	60.5	165.7

## Disclaimer

The views expressed in this article are those of the authors and do not reflect the official policy or position of the United States Air Force, Department of Defense, or the U.S. Government. U.S. Government work not protected by U.S. copyright. This document has been approved for public release.
